# Ascorbic Acid Inhibits Liver Cancer Growth and Metastasis *in vitro* and *in vivo*, Independent of Stemness Gene Regulation

**DOI:** 10.3389/fphar.2021.726015

**Published:** 2021-08-24

**Authors:** Jingjing Wan, Juan Zhou, Lu Fu, Yubin Li, Huawu Zeng, Xike Xu, Chao Lv, Huizi Jin

**Affiliations:** ^1^ School of Pharmacy, Shanghai Jiao Tong University, Shanghai, China; ^2^ School of Pharmacy, Naval Medical University, Shanghai, China; ^3^ Institute of Interdisciplinary Integrative Medicine Research, Shanghai University of Traditional Chinese Medicine, Shanghai, China

**Keywords:** ascorbic acid, cancer stem cells, metastasis, stemness genes, H_2_O_2_, apoptosis

## Abstract

Experimental and clinical evidence has indicated that the natural product ascorbic acid (AA) is effective in preventing and treating various types of cancers. However, the effect of AA on liver cancer metastasis has not yet been reported. Cancer stem cells (CSCs) play pivotal roles in cancer metastasis. Here, we demonstrated that AA selectively inhibited the viability of both liver cancer cells and CSCs, reduced the formation of cancer cell colonies and CSC spheres, and inhibited tumor growth *in vivo*. Additionally, AA prevented liver cancer metastasis in a xenotransplantation model without suppressing stemness gene expression in liver CSCs. Further study indicated that AA increased the concentration of H_2_O_2_ and induced apoptosis in liver CSCs. Catalase attenuated the inhibitory effects of AA on liver CSC viability. In conclusion, AA inhibited the viability of liver CSCs and the growth and metastasis of liver cancer cells *in vitro* and *in vivo* by increasing the production of H_2_O_2_ and inducing apoptosis. Our findings provide evidence that AA exerts its anti-liver cancer efficacy *in vitro* and *in vivo*, in a manner that is independent of stemness gene regulation.

## Introduction

One of the main causes of cancer-related death is distant metastasis that occurs in cancer patients, and cancer stem cells (CSCs) are an important driving force for cancer metastasis. CSCs, also referred to as tumor-initiating cells, have a stronger tumor-forming ability than somatic or non-tumorigenic cancer cells (Ponti et al., 2005; Ma et al., 2007). CSCs play key roles in the development of metastasis in multiple cancers. In colorectal cancer, CD26^+^ CSCs caused distant metastasis when injected into the mouse cecal wall, while the presence of CD26^+^ CSCs in primary tumors can predict distant metastasis in cancer patients (Pang et al., 2010). Also, Lgr5^+^ or CD44v6^+^ CSCs are required for the generation of metastatic tumors (Todaro et al., 2014; De Sousa e Melo et al., 2017). In squamous cell carcinoma of the head and neck, it was observed that BMI1^+^ CSCs regulated the invasive growth and cervical lymph node metastasis in a mouse model (Chen D. et al., 2017). A recent study at the single-cell level in breast cancer has shown that early-stage metastatic cells possess a distinct stem-like gene expression signature (Wylie et al., 2015).

Liver cancer is a heterogeneous disease, and liver CSCs play important roles in the development of this disease. Inhibition of ICAM-1, a marker of hepatocellular CSCs, suppresses tumor formation and metastasis in mice (Liu et al., 2013). All-trans retinoic acid can effectively induce the differentiation of CSCs, and it also enhances the cytotoxicity of cisplatin and increases the inhibition of hepatocellular carcinoma (HCC) cell migration *in vitro* and metastasis *in vivo* in combination with cisplatin (Zhang et al., 2013). All of these studies have demonstrated a key role for CSCs in cancer metastasis and suggested that CSCs are a promising target for developing effective therapeutic agents that can be used to treat metastatic cancer.

The natural product ascorbic acid (AA) is an important water-soluble vitamin and is one of the early unorthodox therapies that has long been used in the field of alternative and complementary medicine for cancer treatment, with profound safety and anecdotal efficacy (Du et al., 2010; Chen et al., 2015). Many clinical and laboratory studies have revealed its effects on cancer prevention and treatment. AA inhibits the growth of prostate, ovarian, and pancreatic cancer cells and neuroblastoma cells. (Maramag et al., 1997; Carosio et al., 2007; Chen et al., 2008; Du et al., 2010; Yun et al., 2015; Schoenfeld et al., 2017). Cameron et al. demonstrated in the 1970s that there was a potential survival benefit for patients who received oral and intravenous administration of AA (Cameron and Pauling, 1976; Cameron and Pauling, 1978). However, two clinical studies performed at the Mayo Clinic have shown no significant difference between oral ascorbate-treated and placebo-treated patients (Moertel and Fleming, 1985; Creagan et al., 1979).

Additional research has shown that oral ingestion of high doses of AA rarely induce a plasma concentration greater than 200 μM, due to the limited absorption and renal excretion. By contrast, both intravenous (i.v.) and intraperitoneal (i.p.) administration of ascorbate result in pharmacologic serum ascorbate concentrations up to 20 mmol/L (Reczek and Chandel, 2015; Verrax and Calderon, 2009). Subsequent studies have shown that high-dose intravenous administration of AA alleviates symptoms and prolongs survival in patients with advanced cancer (Cameron and Pauling, 1976; Cameron and Pauling, 1978; Cameron and Campbell, 1974; Padayatty et al., 2006; Raymond et al., 2016). AA also significantly reduces the metastasis of B16FO melanoma cells injected into mice who were deficient in AA and unable to synthesize it (Cha et al., 2013). However, there have been no reports describing the effects of AA on liver cancer metastasis.

With the participation of transition metals (such as copper and iron), a high dose of AA as an electron donor produces extracellular ascorbate anion and H_2_O_2_, which play important roles in AA-induced anticancer activity (Chen et al., 2015). H_2_O_2_, an important reactive oxygen species (ROS), plays numerous roles in cancer cells, where a low concentration of H_2_O_2_ is involved in various signal transduction and cell functions, and a high concentration of H_2_O_2_ causes DNA damage and promotes cell apoptosis. Du et al*.* demonstrated that AA decreases the clonogenic survival of pancreatic cancer cell lines, while treatment of cells with H_2_O_2_ scavengers can reverse AA’s anticancer activity (Du et al., 2010). Chen et al. reported that AA causes significant cytotoxicity in cancer cells, while glutathione reduces the cytotoxicity by attenuating AA-induced H_2_O_2_ production (Chen et al., 2005; Chen et al., 2011).

In this study, we investigated the inhibitory effects of AA on liver cancer cells and liver CSCs *in vitro* and *in vivo*. We found that AA inhibited the growth and metastasis of liver cancer cells and liver CSCs, although AA also increased the expression levels of stemness genes. Further molecular mechanism studies indicated that the increased concentration of H_2_O_2_ and the enhanced apoptosis by AA play vital roles in its efficacy against liver cancer.

## Materials and Methods

### Cell Culture

Human liver cancer cell lines Huh7 and Hep3B and normal human liver cell line L02 cells were cultured in Dulbecco’s modified Eagle’s medium (DMEM) containing 10% fetal bovine serum (FBS) and 1% penicillin/streptomycin. Huh7 and Hep3B CSCs were enriched and maintained on poly-HEMA coated plates in serum-free DMEM/Nutrient Mixture F-12 (F-12) medium containing 20 ng/ml epidermal growth factor (EGF) (236-EG-200, R&D Systems), 10 ng/ml fibroblast growth factor (FGF) (233-FB-025, R&D Systems), and 1% penicillin/streptomycin (Pang et al., 2010; Li et al., 2015). For preparing poly-HEMA coated plates, 6-well plates were pre-coated with 1.2% (w/v) poly-HEMA (Re et al., 1994).

### Detection of Cell Viability

Cell viability was measured by Cell Counting Kit-8 (CCK-8) (Dojindo Laboratories) according to the user’s manual. The cell viability in each group is expressed as the percentage of untreated control cell viability (Wu et al., 2017).

### Flow Cytometric Analysis

To examine the expression of CD133 and CD44, Huh7 and Hep3B stem cells were digested with 0.05% trypsin. Next, 10^6^ cells/100 μl of single cells were resuspended and incubated with PE-labeled CD133 (1:50, Miltenyi Biotec) or CD44 (1:50, Miltenyi Biotec) in the dark for 15 min, washed twice with cold phosphate-buffered saline (PBS), resuspended in 400 μl PBS, and analyzed using flow cytometry (Becton Dickinson FACS Vantage SE, San Jose, CA, United States).

To analyze cell apoptosis, Huh7 stem cells were digested with 0.05% trypsin. Then, 1 × 10^6^ single cells were resuspended and mixed with 10 μl Annexin V-fluorescein isothiocyanate (FITC, 130-097-928, Miltenyi Biotec), incubated in darkness for 15 min, washed with 1 ml 1× Annexin V Binding Buffer and resuspended in 500 μl 1× Annexin V Binding Buffer, mixed with propidium iodide (PI) solution, and then analyzed by flow cytometry (Cheng et al., 2017).

### RNA Isolation and Quantitative Real-Time PCR

Total RNA was isolated using a Tissue RNA Kit (R6311-01, Biomiga). RNA (1 μg) was reverse-transcribed into cDNA using GoScript Reverse Transcriptase (A5001, Promega). Quantitative real-time PCR was completed using the Power Up SYBR Green Master Mixture (Thermo Fisher) with the StepOne Plus Real-Time PCR System (Thermo Fisher), according to a protocol from a previous study (Wu et al., 2017). Specific primers for *CD90* and *EPCAM* were created according to Luo et al. (2015). Specific primers for *CD133, OCT4 (POU5F1), NANOG, SOX2*, and beta-actin were created according to Ma et al. (2010).

### Animal Experiments

All of the mice were maintained in a pathogen-free facility, and all of the animal experiments were approved by the Committee on the Ethics of Animal Experiments of the Naval Medical University, China. For the animal experiments, 6-week-old female nude BABL/c mice were used, and 2 × 10^6^ Huh7 or Hep3B cells were subcutaneously inoculated into the nude mice (Ma et al., 2018; Yuan et al., 2015). Three weeks later, PBS (control group) or 4 g/kg AA was injected intraperitoneally twice daily for 26 days. The tumor volume was calculated as: total volume = (length × width^2^)/2 (Naito et al., 1986). Lung and liver tissues were fixed with 4% polyformaldehyde, and serial sections (four sections per tissue with a 30-μm step) were created and stained with hematoxylin and eosin (HE) (Cheng et al., 2017).

### Western Blot

Western blot was completed according to a protocol from a previous study (Wu et al., 2017). Briefly, cells or tissues were lysed with Radioimmunoprecipitation Assay (RIPA) Lysis Buffer (P0013C, Beyotime Biotechnology, China) and centrifuged at 13,000 rpm for 15 min. The supernatant was separated by sodium dodecyl sulfate (SDS)-polyacrylamide gel and transferred to a polyvinylidene difluoride (PVDF) membrane. The membrane was incubated overnight with anti-NANOG (1:500, ab109250, Abcam), anti-SOX2 (1:500, ab92494, Abcam, UK), anti-ALDH1A1 (1:1,000, ab52492, Abcam), or anti-β-actin (1:1,000, 3700S, Cell Signaling Technology) primary antibodies, washed with Tris-buffered saline (TBS) containing 0.1% Tween-20 (TBST) three times, incubated with secondary antibody (926-32210, 1:20,000 for β-actin and 926-32211, 1:5,000 for others, LI-COR, Biosciences), and analyzed with the Odyssey Infrared Imaging System (LI-COR, Biosciences).

### Detection of H_2_O_2_

The H_2_O_2_ concentration was measured using a H_2_O_2_ Assay Kit (S0038, Beyotime Biotechnology, China) according to the user’s manual. Simply, 1 × 10^6^ cells were lysed in 200 μl lysis buffer and centrifuged for 5 min at 12,000 rpm. Every 50 μl of the supernatant was mixed with 100 μl of H_2_O_2_ detection reagent and incubated for 30 min at room temperature. Absorbance was determined at 560 nm using an Epoch Microplate Spectrophotometer (BioTek). For catalase experiments, catalase was added prior to AA treatment.

### Sphere Formation Assay and Colony Formation Assay

For the sphere formation experiment, cells were digested into single cells with trypsin. Then, 100 cells/well were plated into a 96-well ultra-low attachment plate and cultured for 2 weeks in serum-free DMEM/F-12 medium containing 20 ng/ml EGF, 10 ng/ml FGF, and AA (0, 0.5, or 1 mM). The number of spheres was counted and photographed.

For the colony formation experiment, 1,000 cells/well were plated into 6-well plates. The colonies were cultured in DMEM containing 10% fetal bovine serum, 1% penicillin/streptomycin, and AA (0, 0.5, or 1 mM). The colonies were then stained with 1% crystal violet.

### Statistical Analysis

Statistical analysis was performed using unpaired *t* tests when comparing two different groups or one-way ANOVA with Tukey’s multiple comparison tests. IC50 values were calculated using Prism software (GraphPad, San Diego, CA, USA) by nonlinear regression to dose-response curves, and expressed as mean and 95% confidence intervals (CI). The data are expressed as the mean ± SEM. *p* < 0.05 was considered statistically significant.

## Results

### AA Selectively Inhibited the Viability of Liver Cancer Cells and Liver CSCs *in vitro*

**FIGURE 1:**
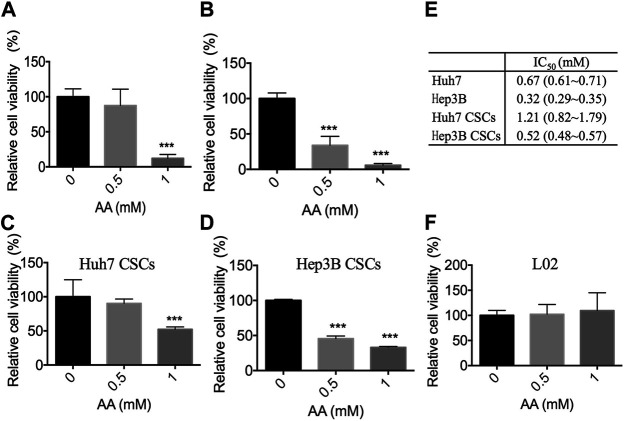
Inhibitory effects of AA on the viability of liver cancer cells *in vitro*. Cells were treated with AA at the concentration as shown and cell viability was measured by CCK-8 assay. **(A)** Cell viability of Huh7 cells. **(B)** Cell viability of Hep3B cells. **(C)** Cell viability of Huh7 CSCs. **(D)** Cell viability of Hep3B CSCs. **(E)** IC50 values of AA, values are mean and 95% confidence. **(F)** Cell viability of L02 hepatocytes. ***p < 0.001.

Two human liver cancer cell lines (Huh7 and Hep3B), the respective CSCs, and a normal human liver cell line L02 were treated with AA at the concentrations of 0, 0.5, or 1 mM, which are easily achievable clinically by intravenous infusion (Chen et al., 2008) (Hoffer et al., 2008). The results showed that AA inhibited the viabilities of liver cancer cells and liver CSCs in a concentration-dependent manner (Figures 1A–D). AA at the concentration of 1 mM decreased the viabilities of Huh7 and Hep3B cells to 12.15 and 5.77%, respectively (Figures 1A,B). For Huh7 and Hep3B CSCs, the viabilities were decreased to 52.37 and 33.04%, respectively, at 1 mM concentration of AA (Figures 1C,D). The IC50 values of AA for Huh7, Hep3B, and Huh7 CSCs and Hep3B CSCs were 0.67, 0.32, 1.21, and 0.52 mM, respectively (Figure 1E). However, AA did not display significant inhibitory effects on the viability of L02 cells at 0.5 mM or 1 mM concentrations (Figure 1F). Together, these data indicated that AA was responsible for selective inhibitory effects on the viabilities of liver cancer cells and liver CSCs.

### AA Inhibits Sphere Formation and Colony Formation in Liver Cancer Cells

**FIGURE 2:**
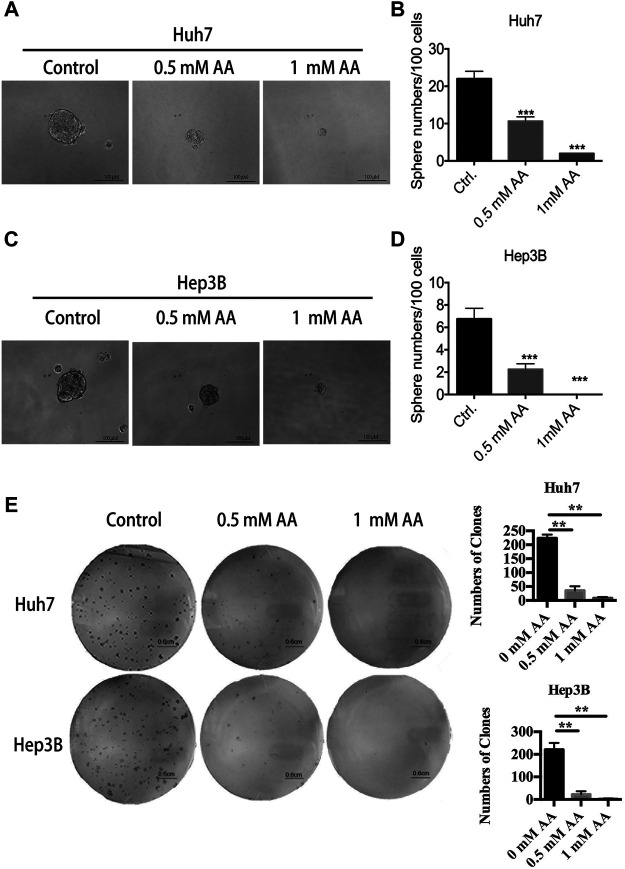
AA reduces sphere formation and colony formation by liver cancer cells. **(A)** Image of spheres formed by Huh7 cells after treatment with AA at indicated concentrations for 14 days. **(B)** Sphere numbers of Huh7 cells after treatment with AA at indicated concentrations for 14 days. **(C)** Image of spheres formed by Hep3B cells after treatment with AA at indicated concentrations for 14 days. **(D)** Sphere numbers formed by Hep3B cells after treatment with AA at indicated concentrations for 14 days. **(E)** Colonies formed by Huh7 or Hep3B cells after treatment with AA at indicated concentrations for 14 days. ***p < 0.001.

We further examined the effects of AA on sphere formation and colony formation. As shown in Figure 2A, AA treatment reduced the volume of spheres formed by Huh7 cells. The number of spheres larger than 50 μm in diameter was markedly decreased in a concentration-dependent manner in AA-treated Huh7 cells (Figure 2B). Twenty-two spheres were formed for every 100 cells in the control group, whereas only two spheres were formed for every 100 cells in the group treated with 1 mM AA. Similar results were obtained for Hep3B cells (Figures 2C,D). As shown in Figure 2E, AA treatment also markedly decreased colony formation in a concentration-dependent manner in Huh7 and Hep3B cell lines. Collectively, our data showed that AA reduced sphere formation and colony formation by liver cancer cells, indicating the inhibitory effects of AA on self-renewal and tumorigenicity of liver cancer cells.

### AA Inhibited Liver Tumor Growth *in vivo*

**FIGURE 3:**
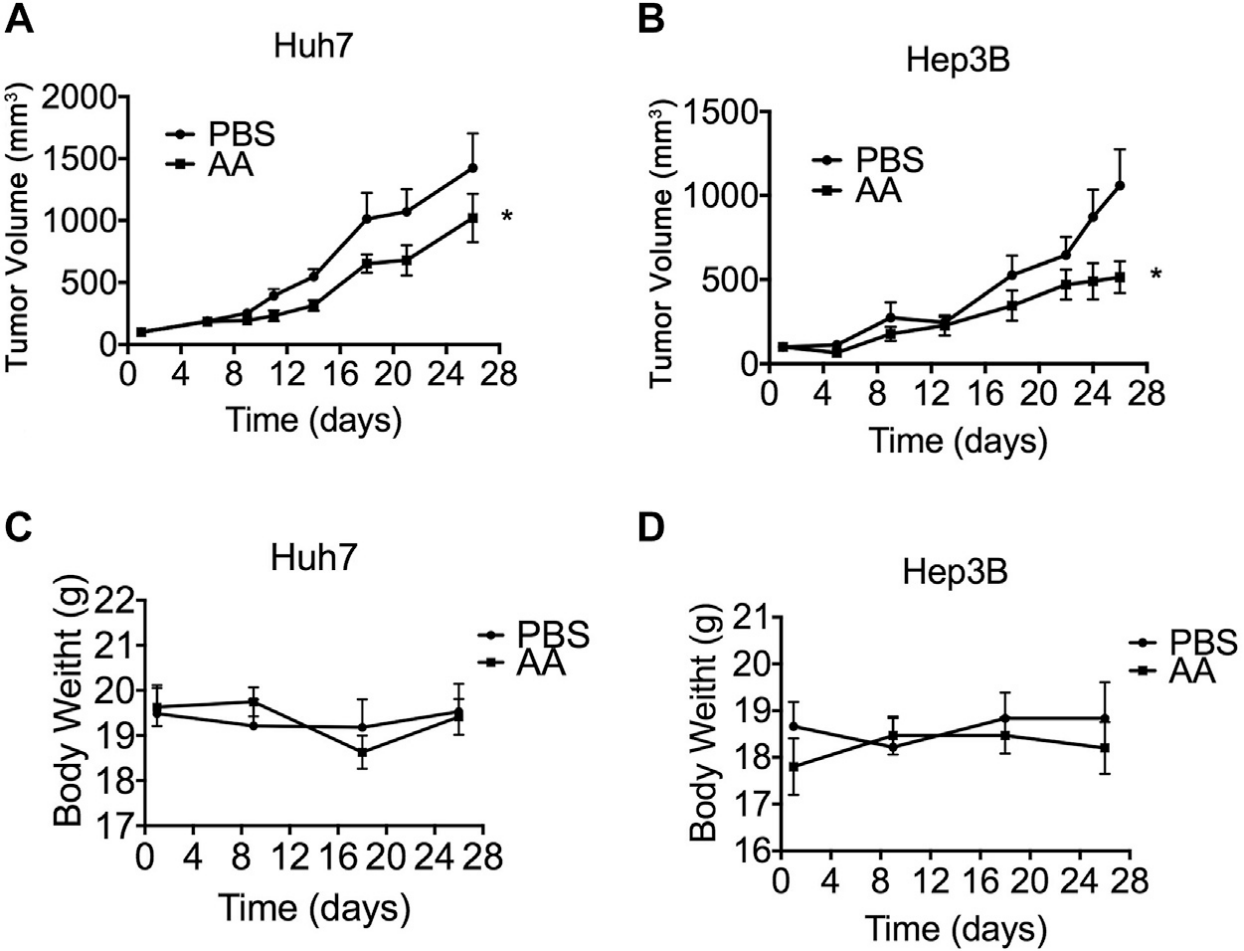
AA suppresses tumor growth *in vivo*. 2 × 10^6^ Huh7 or Hep3B cells were inoculated subcutaneously into nude mice. 3 weeks later, PBS (control group) or 4 g/kg AA was injected intraperitoneally twice daily for 26 days. Tumor volume was measured every 2–3 days and animal body weight was measured every 9 days. **(A)** Volumes of tumors formed by Huh7 cells. **(B)** Volumes of tumors formed by Hep3B cells. **(D)** Body weight of animals engrafted with Huh7 cells. **(D)** Body weight of animals engrafted with Hep3B cells *p < 0.05.

We determined the effects of AA on tumor growth in mice bearing Huh7 and Hep3B xenografts. As mentioned above, AA concentrations in human plasma and cells were tightly controlled. With the oral ingestion of high doses of vitamin C, even at 100 times the recommended dietary allowance, the plasma concentration rarely exceeds 200 µM. Both i.v. and i.p. administration of ascorbate induced pharmacologic serum ascorbate concentrations up to 20 mmol/L. To obtain a pharmacologic serum ascorbate concentration, the i.p. administration method was selected. Compared with the PBS control group, AA treatment significantly suppressed the growth of Huh7 and Hep3B xenograft tumors *in vivo* (Figures 3A,B) without significantly changing the animal’s body weight (Figures 3C,D).

### AA Prevents Tumor Metastasis *in vivo*

**FIGURE 4:**
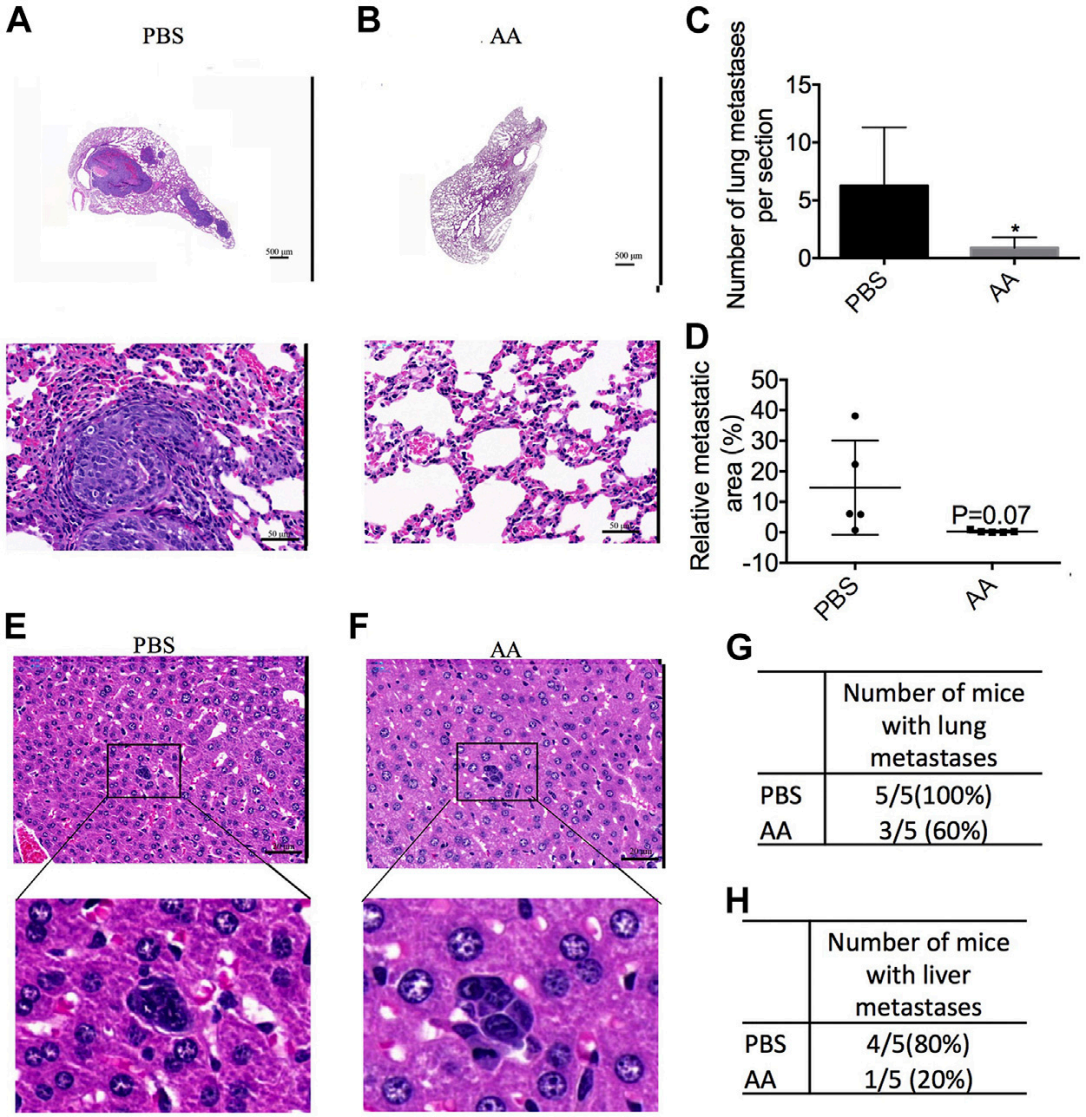
AA prevents tumor metastasis *in vivo*. 2 × 10^6^ Huh7 cells were inoculated subcutaneously into female BALB/c nude mice. After 3 weeks, PBS (control group) or 4 g/kg AA was injected intraperitoneally twice daily. At the end of the experiment, the animals were sacrificed to examine liver and lung metastasis. **(A**,**B)** HE staining of lung section (A: control group, B: AA group; A, B upper: 2× magnification; A, B lower: 40× magnification). **(C**,**D)** the number and area ratio of metastatic tumors in each lung section. **(E**,**F)** HE staining of liver section. (E: control group, F: AA group; 40 × magnification). **(G)** Number of mice with lung metastases in each group. **(H)** Number of mice with liver metastases in each group. *p < 0.05.

As shown in Figures 4A,B, AA-treated mice developed fewer metastatic lung tumors as compared to the control group. The number of metastatic lung tumors in AA-treated mice was 0.90 ± 0.40 (*n* = 5), and that in the control mice was 6.25 ± 2.27 (*n* = 5) (Figure 4C). The area ratio of metastatic lung tumors in AA-treated mice was 0.29 ± 0.17 (*n* = 5), and that in control mice was 14.61 ± 6.91 (*n* = 5) (Figure 4D). The metastatic tumors in the livers of either the control or AA groups were small (Figures 4E,F). In the control group, 5 of 5 mice developed metastatic lung tumors, whereas 3 of 5 mice exhibited metastatic lung tumors in the AA-treated group (Figure 4G). Additionally, in the control group, 4 of 5 mice developed metastatic liver tumors, while in the AA-treated group, 1 of 5 mice developed metastatic liver tumors (Figure 4H). In summary, our data demonstrated that AA treatment reduced liver and lung metastasis of liver cancer cells inoculated subcutaneously into nude mice.

### AA Upregulated the Expression of Stemness Genes in Liver Cancer Cells and Tumors

**FIGURE 5:**
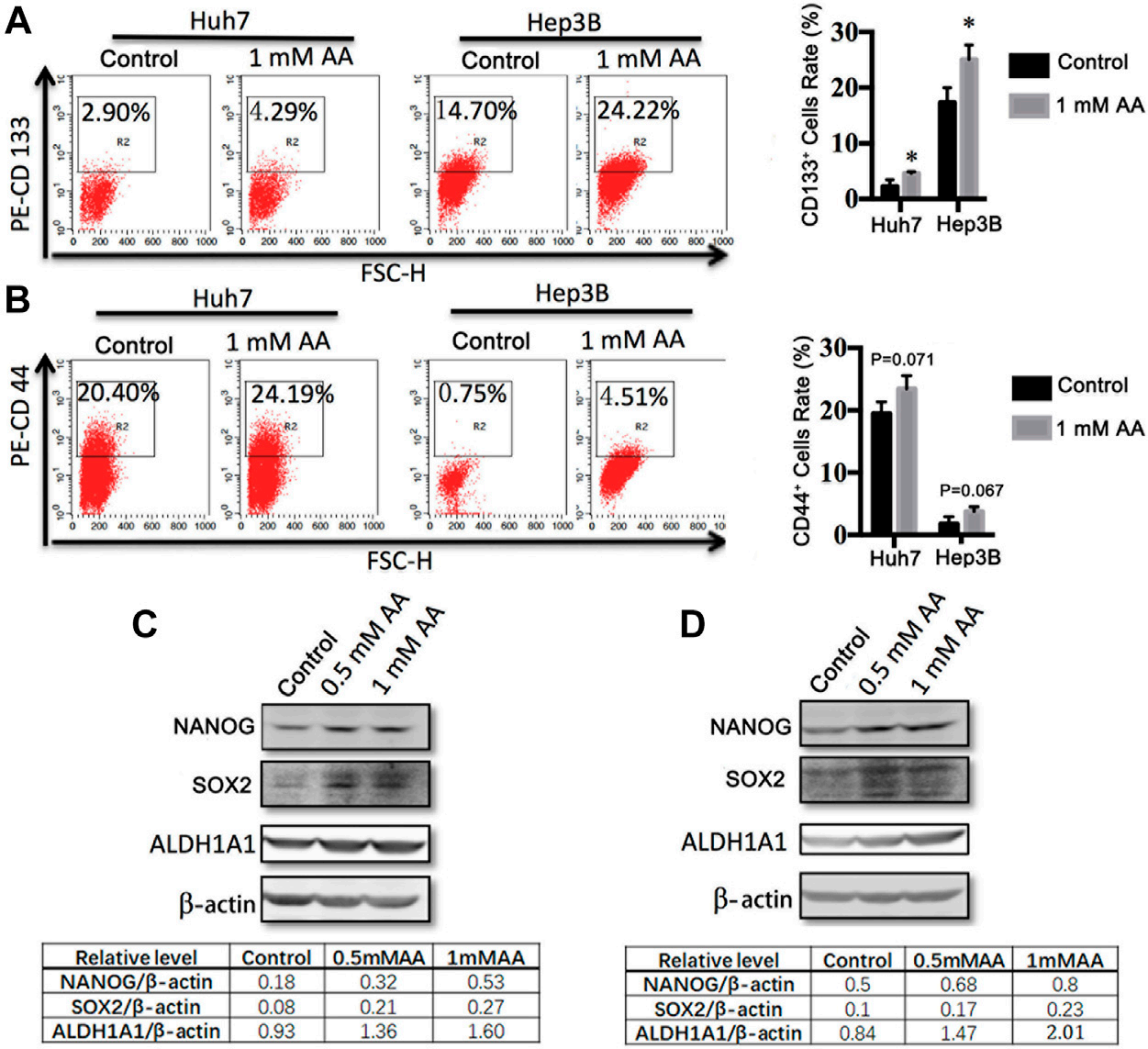
AA regulates the expression of stemness genes in liver cancer cells. **(A,B)** Flow cytometric analysis of the expressions of CD133 and CD44 in Huh7 **(A)** and Hep3B **(B)** stem cells treated with different concentrations of AA. **(C**,**D)** The protein levels of stemness genes in Huh7 **(C)** and Hep3B **(D)** stem cells treated with different concentrations of AA.

We investigated the effects of AA on the expression of stemness genes. Flow cytometric analysis showed that AA treatment increased CD133^+^ cells and CD44^+^ cells in both Huh7-and Hep3B-derived stem cells (Figures 5A,B). CD133 antigen was identified as a CSC marker in various cancer types, including liver cancer. CD44, a transmembrane glycoprotein, is also considered as an important liver CSC marker (Zhu et al., 2010; Yang et al., 2008). For Huh7 CSCs, AA at 1 mM increased CD133^+^ cells and CD44^+^ cells from 2.90 to 14.70%–4.29 and 24.19%, respectively (Figures 5A,B). For Hep3B CSCs, CD133^+^ cells and CD44^+^ cells were increased by AA from 20.40 to 0.75%–24.22 and 4.51%, respectively (Figures 5A,B). Western blot analysis showed that the protein levels of embryonic stem cell markers NANOG and SOX2 as well as liver CSC marker ALDH1A1 were increased after treatment with AA in Huh7-and Hep3B-derived stem cells (Figures 5C,D).

**FIGURE 6:**
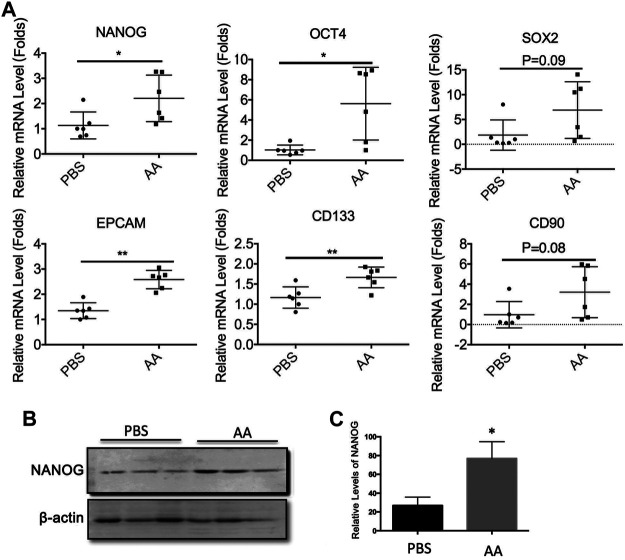
AA regulates the expressions of stemness genes in liver tumors *in vivo*. 2 × 10^6^ Huh7 cells were inoculated subcutaneously into female BALB/c nude mice. Three weeks later, PBS (control group) or 4 g/kg AA were injected introperitoneally twice a day. At the end of treatment, the animals were sacrificed and tumors were collected for analysis. (**A)** mRNA levels of stemness genes in tumor tissue. **(B,C)** Protein levels of NANOG in tumor tissue. *p < 0.05. **p < 0.01.

We also examined the effects of AA on the expression of stemness genes in liver tumors *in vivo*. Consistent with the *in vitro* results, the mRNA expression levels of *NANOG*, *OCT4*, *SOX2*, *EPCAM*, *CD133*, and *CD90* were upregulated in the AA-treated tumors (Figure 6A). Also, the protein level of NANOG was increased in the AA-treated group as compared with that of the control group (Figures 6B,C). Collectively, our data showed that AA upregulated the expression of stemness genes in liver cancer cells *in vitro* and *in vivo*.

### AA Enhanced the Production of H_2_O_2_ and Promoted the Apoptosis of Liver CSCs

**FIGURE 7:**
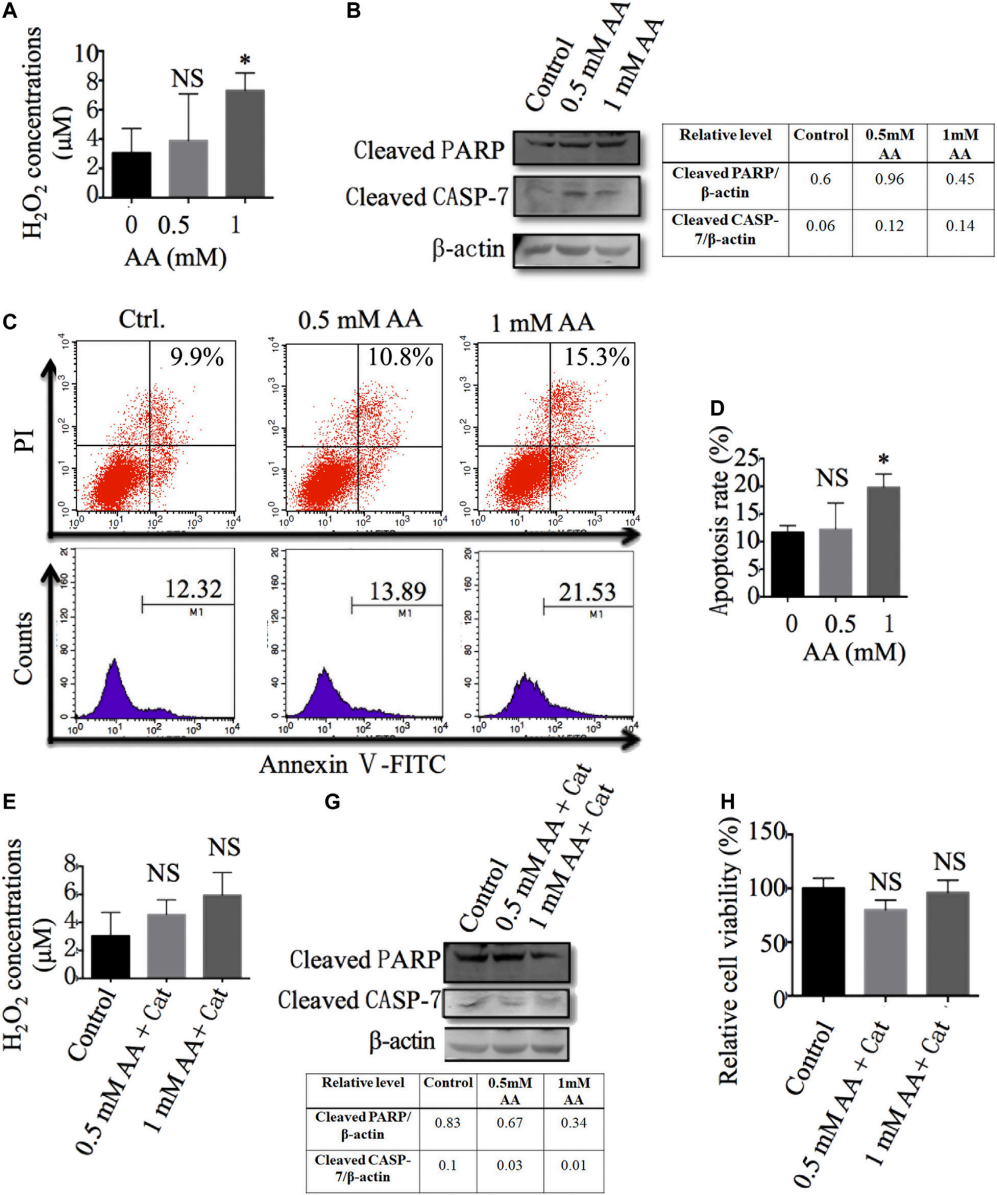
AA reduces liver CSC viability *via* increasing the production of H2O2 and induction of cell apoptosis. **(A)** The content of H_2_O_2_ in Huh7 stem cells treated with different concentrations of AA. **(B)** The protein levels of cleaved PARP and caspase 7 in Huh7 stem cells treated with different concentrations of AA. **(C**, **D)** Apoptosis of Huh7 stem cells treated with different concentrations AA. **(E)** The content of H_2_O_2_ in Huh7 stem cells treated with different concentrations of AA in the presence of 100 μg/ml catalase (Cat: catalase, Sigma -Aldrich). **(F)** The protein levels of cleaved PARP and caspase 7 in Huh7 stem cells treated with different concentrations of AA in the presence of 100 μg/ml catalase. **(G)** Viabilities of Huh7 stem cells treated with different concentrations of AA in the presence of 100 μg/ml catalase. *p < 0.05.

It was reported that H_2_O_2_ plays an important role in AA’s anticancer activity (Lennicke et al., 2015; Chaiswing et al., 2018). To determine the role of H_2_O_2_ in the inhibitory effect of AA on liver CSCs, we first evaluated the concentrations of H_2_O_2_ in Huh7-derived CSCs with or without AA treatment. As shown in Figure 7A, AA treatment increased the concentration of H_2_O_2_ in Huh7-derived CSCs. Furthermore, AA increased the protein levels of cleaved poly (ADP-ribose) polymerase (PARP) and cleaved caspase-7 (Figure 7B) and promoted cell apoptosis (Figures 7C,D).

Catalase, as a specific H_2_O_2_ scavenger, converts the ROS H_2_O_2_ to water and oxygen and thereby mitigates the cytotoxic effects of H_2_O_2_. We also found that the addition of catalase reversed the effects of AA on the production of H_2_O_2_ and the cleavage of PARP and caspase-7 (Figures 7E,F). More importantly, the addition of catalase reduced the inhibitory effects of AA on liver CSC viability (Figure 7G), which was consistent with previous reports describing the dependence of AA’s cytotoxicity on the generation of H_2_O_2_ (Du et al., 2010; Chen et al., 2015; Chen et al., 2005). In conclusion, our data indicate that AA exerts its inhibitory effects on liver CSCs through the production of H_2_O_2_ and the promotion of cell apoptosis.

## Discussion

Various factors lead to an increased risk of liver cancer. Among these factors, it has been reported that alcoholic liver disease is the most common cause of HCC, accounting for approximately 30% of all HCC cases (Morgan et al., 2004). Liver cancer is one of the common causes of cancer-related death. Metastasis and recurrence are the main causes of primary liver cancer-associated mortality. Liver CSCs, possessing a higher migration ability and tumorigenicity, are closely related to metastasis and recurrence of liver cancer. Liver CSCs are considered an important target for liver cancer therapy. For example, WYC-209, a synthetic retinoid, inhibited the proliferation of malignant murine melanoma tumor-repopulating cells and abrogated 87.5% of lung metastases of melanoma tumor-repopulating cells (Chen et al., 2018).

It was reported that AA inhibited the growth of various types of cancer, including colorectal cancer cells, neuroblastoma cells, and ovarian cancer cells. However, its effect on liver cancer metastasis has not yet been reported. Consistent with a previous study (Lv et al., 2018), we found that AA inhibited the viability of liver cancer cells without significantly inhibiting the viability of L02 cells, which are normal human hepatocytes. Furthermore, AA significantly attenuated the viability of liver CSCs and reduced the colony formation ability and sphere formation ability of liver cancer cells *in vitro*, indicating the inhibition by AA on self-renewal and tumorigenicity of liver cancer cells. Because CSCs are involved in important functions in cancer metastasis and AA shows inhibitory effects on liver CSCs, we further examined the effects of AA on liver cancer metastasis. As expected, AA inhibited the metastasis of liver cancer cells to the lung and liver in a subcutaneous xenotransplantation model.

Stemness genes play vital roles in regulating cancer metastasis. In most cases, stemness genes promote cancer metastasis (Lv et al., 2017; Baccelli et al., 2013; Tang et al., 2012; Celià-Terrassa and Kang, 2016). Sox2, a transcription factor involved in the regulation of embryonic development, functions as a novel regulator of cell invasion, migration, and metastasis in several cancer types (Feng and Lu, 2017; Weina and Utikal, 2014). However, it was recently reported that REX1, an embryonic stem cell marker, inhibits liver cancer metastasis, indicating the complex functions of stemness genes in the process of cancer metastasis (Luk et al., 2019). AA regulates the expression of stemness genes, and in human embryonic stem cells, AA caused specific DNA demethylation of 1,847 genes (including the important stem cell genes) (Chung et al., 2010) and also inhibited retinoic acid-induced differentiation of embryonic stem cells (Wu et al., 2014). Furthermore, AA alleviated cell aging and increased the production of induced pluripotent stem cells in mice and human cells (Esteban et al., 2010; Wang et al., 2011).

In adult stem cells, AA enhanced the stemness of mouse corneal epithelial stem cells/progenitor cells and promoted the healing of corneal epithelial injury (Chen J. et al., 2017). AA also reduced stemness gene expression in liver cancer (Lv et al., 2018). Unexpectedly, our data suggested that AA promoted the expression of genes related to cancer stemness. AA increased the production of CD133^+^ and CD44^+^ cells and the protein levels of NANOG, SOX2, and ALDH1A1 *in vitro* and upregulated the mRNA expression levels of *NANOG* and *SOX2* and the protein level of NANOG in Huh7 transplanted tumors. Our data suggest that AA inhibits liver cancer metastasis *via* a pathway independent of stemness gene regulation. However, the detailed mechanisms of AA-induced expression changes of stemness genes require further study.

Our results indicated that AA did not downregulate the expression of stem genes in liver cancer cells, which implies that other mechanisms are involved in the inhibition of liver cancer metastasis by AA. H_2_O_2_ plays an important role in AA’s anticancer activity (Du et al., 2010; Chen et al., 2005; Chen et al., 2011). H_2_O_2_, a key ROS, is involved in cell differentiation, growth, and survival. High levels of H_2_O_2_ can induce cell cycle arrest and apoptosis in cells (Lennicke et al., 2015; Chaiswing et al., 2018). With the participation of transition metals (such as copper and iron), a high dose of AA as an electron donor produces extracellular ascorbate anion and H_2_O_2_. H_2_O_2_ is a cell permeant, and its accumulation induces DNA and mitochondrial damage, and apoptosis of tumor cells. The addition of H_2_O_2_ to tumor cells produced the same cell death effect as that caused by AA, while simultaneous use of the antioxidants N-acetylcysteine or catalase with AA inhibited AA-induced tumor cell death. These results further demonstrate the key role of H_2_O_2_ in AA’s action upon tumor cells. (Chen et al., 2008; Verrax and Calderon, 2009; Chen et al., 2005; Chen et al., 2007). Normal cells exhibit both catalase and glutathione peroxidase activities, which efficiently detoxify H_2_O_2_. This might be the reason why AA selectively inhibited tumor cells, while it had no toxic effects on normal cells (Chen et al., 2005). We examined the changes in the H_2_O_2_ concentration in liver CSCs treated with AA and found that the H_2_O_2_ concentration was increased after AA treatment. AA treatment also increased the protein levels of apoptotic mediators including cleaved PARP and caspase-7 and enhanced the cell apoptosis of CSCs, while the addition of catalase reduced these effects. These results suggest that AA might induce CSC apoptosis by increasing the intracellular concentration of H_2_O_2_.

In conclusion, AA inhibited the viability of CSCs and prevented liver cancer metastasis without reducing the expression of stemness genes in liver cancer cells. The inhibitory effects of AA on liver CSCs can result from the production of H_2_O_2_ and promotion of cell apoptosis. Our findings provide evidence that supports AA as an effective therapeutic agent for liver cancer metastasis and suggest that additional effects other than inhibition of stemness genes may be considered during later evaluation of the effects of AA on CSCs and cancer metastasis.

## Data Availability

The original contributions presented in the study are included in the article/Supplementary Material, further inquiries can be directed to the corresponding authors.
